# Risk Stratification for Organ/Space Surgical Site Infection in Advanced Digestive System Cancer

**DOI:** 10.3389/fonc.2021.705335

**Published:** 2021-11-09

**Authors:** Chen Sun, Hui Gao, Yuelun Zhang, Lijian Pei, Yuguang Huang

**Affiliations:** ^1^ Department of Anaesthesiology, Peking Union Medical College Hospital, Chinese Academy of Medical Sciences & Peking Union Medical College, Beijing, China; ^2^ Medical Research Centre, Peking Union Medical College Hospital, Chinese Academy of Medical Sciences & Peking Union Medical College, Beijing, China; ^3^ Outcomes Research Consortium, Cleveland, OH, United States

**Keywords:** organ/space surgical site infection, risk stratification, advanced digestive system cancer, perioperative management, postoperative complication

## Abstract

**Background:**

Organ/space surgical site infection (organ/space SSI) is a serious postoperative complication, closely related to a poor prognosis. Few studies have attempted to stratify the risk of organ/space SSI for patients with advanced digestive system cancer. This study aimed to identify a simple risk stratification for these patients based on perioperative factors.

**Methods:**

The study was based on two randomized controlled trials (RCT) (NCT02715076, ChiCTR-IPR-17011099), including 839 patients undergoing elective radical resection of advanced digestive system cancer. The primary outcome was organ/space SSI within 30 days after surgery. Multivariable logistic regression model was used to identify risk factors. The risk of organ/space SSI stratified over those risk factors was compared using chi-square tests and the relative risk (RR) was estimated.

**Results:**

Among the 839 patients, 51 developed organ/space SSI (6.1%) within 30 days after surgery. According to the multivariable logistic regression model, 3 procedure types, including gastrectomy (OR=8.22, 95% CI: 2.71-24.87, *P*<0.001), colorectal resection (OR=8.65, 95% CI: 3.13-23.85, *P*<0.001) and pancreatoduodenectomy (OR=7.72, 95% CI: 2.95-20.21, *P*<0.001), as well as anaesthesia time > 4 h (OR=2.38, 95% CI: 1.08-5.27, *P*=0.032) and prolonged ICU stay (OR=4.10, 95% CI: 1.67-10.10, *P*=0.002), were risk factors for postoperative organ/space SSI. The number of risk factors was significantly associated with an increased risk of organ/space SSI (*P*<0.001), which was 2.8% in patients with 0-1 risk factor (RR=0.20, 95% CI: 0.11-0.35), 13.0% in patients with 2 risk factors (RR=3.64, 95% CI: 2.14-6.20) and 35.7% in patients with 3 risk factors (RR=6.41, 95% CI: 3.01-13.65).

**Conclusion:**

This study is a preliminary exploratory and provides a simple risk stratification to identify the risk of postoperative organ/space SSI for patients with advanced digestive system cancer. Further research is needed to validate and generalize the results in a wider population.

**Clinical Trial Registration:**

ClinicalTrials.gov, identifier NCT02715076; Chinese Clinical Trial Registry [https://www.chictr.org.cn/enindex.aspx], identifier ChiCTR-IPR-17011099.

## Introduction

Surgical site infection (SSI) is one of the most common complications in surgical patients and is closely related to poor prognosis. The incidence of SSI in major surgery can reach 10-20% (1–3). In the United States, SSI causes more than 90,000 readmissions, extends the average length of hospital stay by 9.7 days, and increases medical costs by 700 million dollars each year (4).

SSI can be classified as superficial SSI, deep SSI and organ/space SSI (1). With the development and popularity of laparoscopic surgery and bloodless surgery, as well as other preventive methods such as hand hygiene, skin disinfection and perioperative prophylactic antibiotics (5–7), the incidence of superficial SSI has dropped by approximately 50% (5). However, organ/space SSI, whose risk factors are quite different from superficial SSI, is still at high risk and has gradually become the major type of SSI, causing unscheduled reoperation, unscheduled readmission, permanent disability and even death (4).

Surgery is the primary treatment for patients with advanced digestive system cancer. Compared with benign diseases, these patients have a higher risk of organ/space SSI. However, previous research on SSI in these patients has focused mainly on superficial SSI (8); only a few studies have investigated the risk factors for organ/space SSI (9–13), concentrated mainly on preoperative factors and not including intraoperative and early postoperative factors. Moreover, the strategies for preventing SSI are increasingly complex and diverse, adding to difficulties in their clinical application. In this study, we intended to provide a simple risk stratification, including not only preoperative factors but also intraoperative and early postoperative factors, for patients with advanced digestive system cancer to help doctors to identify the risk of postoperative organ/space SSI.

## Materials and Methods

### Study Population

This study was based on two randomized controlled trials (NCT02715076 (14) and ChiCTR-IPR-17011099, http://www.chictr.org.cn/showproj.aspx?proj=18892) in Peking Union Medical College Hospital (PUMCH). Participants undergoing elective radical resection of advanced digestive system cancer between February 1, 2016, and January 31, 2019 in these two RCTs were included in our study. According to the exclusion criteria of the two RCTs, patients who suffered infectious diseases or had an axillary temperature > 37.5°C within 4 weeks before surgery, had clinically important coagulopathy in the judgement of the attending anaesthesiologist, had an end-stage renal disease requiring dialysis, had a body mass index (BMI) exceeding 30 kg/m^2^ or were diagnosed by the surgeon to be at particular infection risk were excluded.

### Perioperative Management

Patients undergoing elective radical resection of advanced digestive system cancer in PUMCH had a uniform diagnosis and treatment procedure. Patients’ preoperative conditions were optimized 2 weeks before surgery, including thorough consultation and treatment, preventive rehabilitation, physical exercise, nutrition enhancement, etc. Advanced cancer was evaluated according to the endoscopic pathological diagnosis and medical imaging examination results. If necessary, neoadjuvant chemotherapy and radiotherapy (1-3 courses of treatment) were performed to shrink the tumor volume, make its boundary clear, potentially decrease intraoperative blood loss, and reduce the risk and difficulty of surgery. The complications of chemotherapy and radiotherapy were corrected at the same time. Finally, after adequate preoperative preparation, elective surgery was performed. 30 minutes before surgery, patients were all treated with prophylactic antibiotics. Generally, it was cefuroxim 1.5g, but it would be replaced by clindamycin if the patient was allergic to penicillin.

After surgery, patients would be indwelled drainage tube. The drainage fluid quantity and traits, as well as other details such as whether the drainage tube is obstructed, were observed daily. When patients recovered well, without bleeding or fistula, and didn’t have any special manifestation after resuming oral intake, the drainage tube would be removed. Generally, the drainage tube was removed 3-5 days after surgery in patients with good recovery. However, in patients undergoing oesophagectomy or pancreatoduodenectomy, it would take a little bit longer time. Surgeons and anesthesiologists would decide whether patients to return to the ward or intensive care unit (ICU) based on their physical conditions. When mechanical ventilation and vasopressors were successfully withdrawn, tissue perfusion was normal, required monitoring and medical care could be safely provided in the general surgical ward, the patients would be transported from ICU to wards.

### Data Collection

Baseline characteristics were collected from the datasets of the two RCTs, including demographics, preoperative variables (BMI, smoking history, American Society of Anaesthesiologists (ASA) classification, Charlson comorbidity index, comorbidities, neoadjuvant chemotherapy and radiotherapy, preoperative parenteral nutrition, prophylactic antibiotics and the most recent preoperative Hb, Hct, Alb, SCr and Glu), operative variables (procedure approach, procedure type, intraoperative blood loss, intraoperative red blood cell (RBC) transfusion, anesthesia time and final core temperature), and early postoperative variables (ICU stay).

To follow the clinical habits and increase the clinical practicability, we set thresholds for certain continuous variables, such as age, ASA classification, intraoperative blood loss, intraoperative RBC transfusion, anesthesia time and final core temperature. The thresholds were selected according to the population median and previous research. ICU stay was classified into no ICU stay (back to ward after surgery), overnight ICU stay (within 24 hours in ICU after surgery) and prolonged ICU stay (> 24 hours in ICU after surgery).

### Outcomes

The primary outcome was organ/space SSI within 30 days after surgery, which was respectively the part of primary outcome (ChiCTR-IPR-17011099) and one of the secondary outcomes (NCT02715076) of two RCTs. We used criteria proposed by the Centers for Disease Control and Prevention (CDC) to define organ/space SSI. Patients who had at least 1 of the following within 30 days after surgery were diagnosed with an organ/space SSI: a. purulent drainage from a drain placed through a surgical incision into the organ/space; b. organisms isolated from an aseptically obtained culture of fluid or tissue in the organ/space; c. an abscess or other evidence of infection involving the organ/space that is found on direct examination, during reoperation, or by histopathologic or radiologic examination; and d. diagnosis of an organ/space SSI by a surgeon or attending physician (1). Suspected cases were confirmed after discussion with an expert panel composed of specialists in anesthesiology, critical care medicine, surgery, and infectious diseases.

Secondary outcomes included superficial SSI within 30 days after surgery, unscheduled reoperation, unscheduled readmission and length of hospital stay. Superficial SSI was also diagnosed according to criteria proposed by CDC (1). Unscheduled reoperation was defined as the need for unplanned second surgery for various reasons within 30 days after surgery, and unscheduled readmission was an emergency admission after discharge for various reasons within 30 days after surgery.

Outcomes observation and follow-up information were obtained from the datasets of those two RCTs, which was collected prospectively. Patient withdrawal was 0 and the 30-day follow-up rate was 100%.

### Statistical Analysis

Descriptive statistics illustrated the frequency of specific baseline characteristics; this was expressed in outcome percentages for dichotomous variables and means or medians with standard deviations and interquartile rates, respectively, for continuous variables.

Binary logistic regression analysis was used in both univariable and multivariable analyses. The area under the receiver operating characteristic curve (AU-ROC) and Hosmer-Lemeshow test were used to assess the discrimination and calibration of the model. Variable selection in the multivariable analysis was based on clinical experience, the results of univariable analysis, participant characteristics, multicollinearity diagnosis and previous research. Variables with a *P* value less than 0.1 were selected preliminarily according to the univariable analysis. Because of the comprehensive consultation and treatment before elective surgery at PUMCH, most participants in this study had normal ranges of some preoperative variables (such as Hb, Hct and Alb). Therefore, considering the limited clinical significance of their association with organ/space SSI, these variables were not included in the multivariable analysis. For multicollinearity diagnosis, the correlation coefficient, variance inflation factor (VIF) and characteristic root system were used. When two or more variables showed collinearity, the choice of which variable was included in the multivariable analysis was based on clinical experience.

Subsequently, to identify a simple risk stratification, associations between the number of risk factors (0-1, 2 or 3) revealed in the multivariable analysis and organ/space SSI risk were measured using chi-square tests. Additionally, the relative risk (RR) and corresponding 95% confidence interval (CI) using the remaining population as the reference group were assessed (RR = risk of SSI in patients with N risk factors/risk of SSI in the remaining population).

All statistical analyses were performed using SPSS 25.0. A 2-sided *P* value less than 0.05 was considered the threshold for statistical significance.

### Ethics

The study was based on two RCTs (NCT02715076, ChiCTR-IPR-17011099), both approved by the ethics committee of Peking Union Medical College Hospital (HS-1019, HS-1121). Written informed consent was obtained from all subjects involved. All methods were carried out in accordance with institutional guidelines and regulations.

## Results

### Baseline Characteristics

A total of 839 patients receiving elective radical resection of advanced digestive system cancer were included in this study, of which 575 were males (68.5%) and 264 were females (31.5%). Among all the patients, 51 (6.1%) developed organ/space SSI within 30 days after surgery, almost all of whom had intra-abdominal infection, and 8 (1.0%) developed sepsis related to organ/space SSI. In terms of secondary outcomes, 17 (2.0%) patients developed superficial SSI, 19 (2.3%) underwent unscheduled reoperation, and 13 (1.5%) experienced unscheduled readmission. The median length of hospital stay was 14 days. Details are shown in **Tables 1**, **2**.

**Table 1:** Baseline characteristics and risk factors for organ/space surgical site infection (Univariable Analysis) in advanced digestive system cancer.

Risk Factor	Overall (n=839)	Univariable analysis	
		Odds Ratio (95% CI)	*P* value
Sex			
Male	575 (68.5)	0.76 (0.42-1.36)	0.360
Female	264 (31.5)		
Age (y)	62.9±10.8		
< 65	432 (51.5)		
≥ 65	407 (48.5)		
BMI (kg/m^2^)	23.5±3.1	1.05 (0.96-1.15)	0.255
Current smoking	255 (30.4)	1.16 (0.63-2.11)	0.638
ASA classification			
I-II	535 (63.8)		
≥ III	304 (36.2)	0.96 (0.53-1.73)	0.885
Charlson comorbidity index			
0	0		
1	0		
2	80 (9.5)	/	0.983
3	137 (16.3)	0.81 (0.25-2.64)	0.723
4	282 (33.6)	0.96 (0.34-2.69)	0.942
5	255 (30.4)	1.00 (0.36-2.83)	0.994
6	85 (10.1)	1.14 (0.33-3.89)	0.835
Comorbidities			
Hypertension	365 (43.5)	1.17 (0.66-2.06)	0.598
Diabetes	183 (21.8)	1.39 (0.73-2.62)	0.316
Coronary heart disease	97 (11.6)	0.82 (0.32-2.12)	0.686
COPD	14 (1.7)	0.94 (0.12-7.36)	0.958
Neoadjuvant chemotherapy	122 (14.5)	0.35 (0.11-1.15)	0.083
Neoadjuvant radiotherapy	57 (6.8)	0.54 (0.13-2.30)	0.407
PN	283 (33.7)	2.34 (1.32-4.13)	0.003
Prophylactic antibiotics	839 (100.0)		
Cefuroxim	803 (95.7)	1.11 (0.26-4.73)	0.893
Clindamycin	36 (4.3)		
Laboratory evaluation			
Hb (g/l)	133.0±18.4	0.99 (0.97-1.00)	0.043
Hct (%)	39.6±4.8	0.94 (0.89-0.99)	0.024
Alb (g/dl)	41.4±4.2	0.95 (0.88-1.02)	0.127
SCr (μmol/l)	74.9±30.8	1.00 (0.98-1.01)	0.589
Glu (mmol/l)	5.8±1.7	0.99 (0.84-1.18)	0.924
Procedure approach			
Open*	468 (55.8)		
Laparoscopic	371 (44.2)	1.34 (0.76-2.35)	0.317
Procedure type**			
Oesophagectomy	120 (14.3)	0.11 (0.02-0.82)	0.031
Gastrectomy	83 (9.9)	1.77 (0.80-3.90)	0.098
Colorectal resection	236 (28.1)	1.56 (0.87-2.82)	0.087
Pancreatoduodenectomy	133 (15.9)	3.51 (1.92-6.41)	<0.001
Hepatic resection	249 (29.7)	0.30 (0.13-0.71)	0.006
Others***	26 (3.1)	0.61 (0.08-4.60)	0.632
Intraoperative blood loss (ml)	152 (50, 390)		
≤500	723 (86.2)		
>500	116 (13.8)	2.55 (1.33-4.87)	0.005
Intraoperative RBC transfusion (U)	163 (19.4)		
≤2	75 (8.9)		
>2	88 (10.5)	1.92 (0.90-4.10)	0.090
Anaesthesia time (h)	4 (3.1, 5.4)		
≤4	419 (49.9)		
>4	420 (50.1)	2.08 (1.15-3.80)	0.016
Final core temperature (°C)	36.3±0.8		
<36.0	330 (39.3)	0.76 (0.42-1.38)	0.363
≥36.0	509 (60.7)		
No ICU stay	633 (75.4)		
Overnight ICU stay (≤24 h)	166 (19.8)	3.51 (2.00-6.23)	<0.001
Prolonged ICU stay (>24 h)	40 (4.8)	4.40 (1.91-10.12)	<0.001

Results presented as $\bar{x}\pm s$ or median (P_25_, P_75_) or n (%).

*Cases in which a laparoscopic procedure was converted to an open procedure were considered open procedures.

**Several patients received different procedure types at the same time.

***Other procedure types included cholecystectomy, choledochotomy and distal pancreatectomy.

CI, confidence interva; BMI, body mass index; COPD, chronic obstructive pulmonary disease; ASA, American Society of Anesthesiologists; PN, parenteral nutrition; Hb, haemoglobin; Hct, haematocrit; Alb, albumin; SCr, serum creatinine; Glu, glucose; RBC, red blood cell; ICU, intensive care unit.

**Table 2:** Outcome characteristics in advanced digestive system cancer.

	Overall (n=839)
Primary outcome Organ/space SSI	51 (6.1)
Intra-abdominal infection	49 (5.8)
Other organ/space SSI	4 (0.5)
Sepsis related to organ/space SSI	8 (1.0)
Secondary outcomes	
Superficial SSI	17 (2.0)
Unscheduled reoperation	19 (2.3)
Unscheduled readmission	13 (1.5)
Length of hospital stay (d)	14 (12, 19)

Results presented as median (P_25_, P_75_) or n (%).

SSI, surgical site infection.

### Risk Factors for SSI

**Table 1** shows the results of univariable analysis of organ/space SSI, in which neoadjuvant chemotherapy, parenteral nutrition, preoperative Hb and Hct, 5 main procedure types, intraoperative blood loss > 500 ml, intraoperative RBC transfusion > 2 U, anesthesia time > 4 h and prolonged ICU stay had a *P* value < 0.1. However, since the preoperative Hb and Hct levels for most patients in our study were normal, they were not included in the multivariable analysis. A diagnosis of multicollinearity suggested obvious collinearity between intraoperative blood loss and intraoperative RBC transfusion. As blood transfusion is often used as a treatment after massive blood loss during surgery for patients without preoperative anemia, we excluded intraoperative RBC transfusion > 2 U.

**Figure 1:**
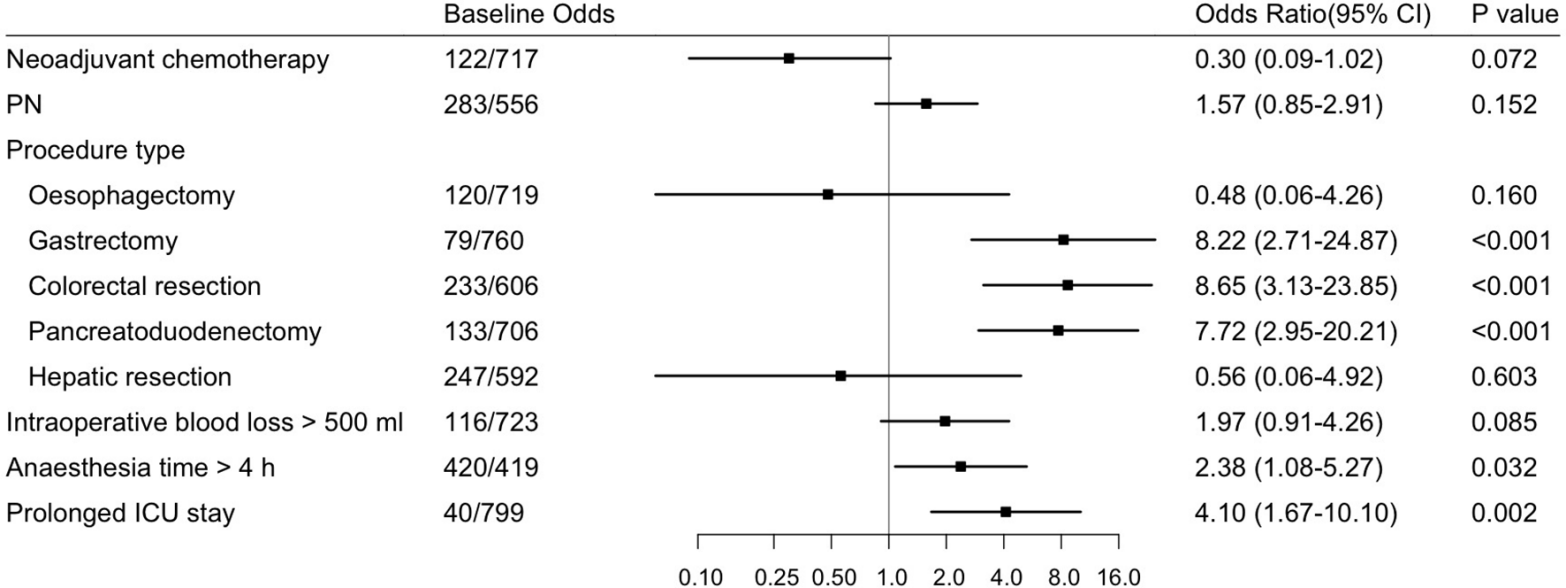
Forest Plot about Risk Factors for Organ/Space Surgical Site Infection (Multivariable Analysis) in Advanced Digestive System Cancer. Odds ratios and 95% CIs are shown, derived from the multivariable logistic regression model (Hosmer-Lemeshow test: *P*=0.881). CI, confidence interval; PN, parenteral nutrition.

**Figure 2:**
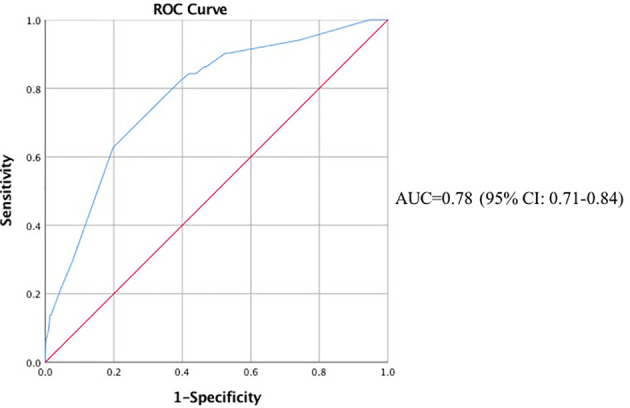
ROC Curve of the Multivariable Analysis of Organ/Space Surgical Site Infection. ROC, receiver operating characteristic; AUC, area under the curve; CI, confidence interval.

Finally, we incorporated neoadjuvant chemotherapy, parenteral nutrition, 5 main procedure types, intraoperative blood loss > 500 ml, anesthesia time > 4 h and prolonged ICU stay into the multivariable analysis. According to the results shown in **Figures 1**, **2**, gastrectomy (OR=8.22, 95% CI: 2.71-24.87, *P*<0.001), colorectal resection (OR=8.65, 95% CI: 3.13-23.85, *P*<0.001), pancreatoduodenectomy (OR=7.72, 95% CI: 2.95-20.21, *P*<0.001), anesthesia time > 4 h (OR=2.38, 95% CI: 1.08-5.27, *P*=0.032) and prolonged ICU stay (OR=4.10, 95% CI: 1.67-10.10, *P*=0.002) were significantly associated with an increased risk of organ/space SSI. The discrimination and calibration of the model were acceptable (AUC was 0.78, 95% CI: 0.71-0.84. Hosmer-Lemeshow test: *P* = 0.881).

### Risk Stratification for SSI

According to the multivariable analysis, 3 risk factors (procedure type, anesthesia time > 4 h and prolonged ICU stay) can be used to stratify postoperative organ/space SSI. The number of risk factors was significantly associated with an increased risk of organ/space SSI (*P*<0.001). Compared with the overall population, patients with 1 or fewer risk factors had a lower risk, while patients with 2 or more risk factors had a significantly higher risk. The risk of organ/space SSI was 2.8% in patients with 0-1 risk factor (RR=0.20, 95% CI: 0.11-0.35), 13.0% in patients with 2 risk factors (RR=3.64, 95% CI: 2.14-6.20) and 35.7% in patients with 3 risk factors (RR=6.41, 95% CI: 3.01-13.65). The association between the number of risk factors and the risk of organ/space SSI is shown in **Table 3**.

**Table 3:** Association of the Number of Risk Factors with the Risk of Organ/Space Surgical Site Infection in Advanced Digestive System Cancer.

Risk factors	0-1	Overall	2	3	*P* value
No. of patients	602	839	223	14	NA
Risk of SSI (%)	2.8	6.1	13.0	35.7	<0.001
Relative risk (95% CI)	0.20 (0.11-0.35)	1	3.64 (2.14-6.20)	6.41 (3.01-13.65)	NA

NA, not applicable; SSI, surgical site infection; CI, confidence interval.

## Discussion

Organ/space SSI is one of the most serious postoperative complications and is closely related to poor prognosis. Although the incidence of superficial SSI has decreased as a result of many preventive methods, the incidence of organ/space SSI is still high in patients undergoing surgical treatment. In this study, the incidence of postoperative organ/space SSI reached 6.1%, 3 times more than that of superficial SSI.

We found that 3 high-risk procedure types (gastrectomy, colorectal resection and pancreatoduodenectomy), long anesthesia time and prolonged ICU stay were significantly associated with an increased risk of organ/space SSI through the multivariable logistic regression model, of which the discrimination and calibration were acceptable. According to our results, for patients whose preoperative condition is well controlled before elective surgery, the identification of operative risk factors will have more clinical significance. At present, surgical wound classification and “major surgery” are still used to identify high-risk surgery of postoperative SSI, both of which are not accurate enough and have limited clinical practicability. In this study, we included different types of digestive system “major surgery” all with class II surgical wound, and found that there were still differences in the risk of postoperative organ/space SSI between them. Gastrectomy, colorectal resection and pancreatoduodenectomy were high-risk surgeries, while oesophagectomy and hepatic resection were not. We hope to help clinicians have a further understanding with “high-risk surgery” of postoperative SSI, not only confined to the surgical wound classification. We should pay more attention to patients receiving gastrectomy, colorectal resection and pancreatoduodenectomy, and multiple approaches are needed to optimize the surgery process, shorten the anesthesia time, reduce surgical trauma, and promote early recovery after surgery. The prolonged ICU stay was defined as staying more than 24 hours in ICU in our study, whereas the majority of organ/space SSIs often occurred between 3 and 7 days after surgery. Our results indicated that for those patients with ICU stay longer than 24 hours and still not be able to be discharged, more attention should be paid to prevent the postoperative organ/space SSI.

Although neoadjuvant chemotherapy did not show a statistically significant difference, it showed a protective effect on postoperative organ/space SSI to a certain extent. This result demonstrated that for patients with advanced digestive system cancer, in cases of anemia, low albumin and other chemotherapy complications were corrected, necessary neoadjuvant chemotherapy could shrink the tumor volume and reduce the difficulty of surgery and the risk of organ/space SSI.

Risk stratification suggested that patients with 1 risk factor or fewer were found to be 5 times less likely than the remaining population to develop organ/space SSI. Conversely, patients with all 3 risk factors were more than 6 times more likely to develop organ/space SSI. This risk stratification provides a simple method to identify high-risk patients with postoperative organ/space SSI. Using this method, patients who may have a significantly greater risk could benefit from more frequent monitoring and obtain a timely response.

In contrast with previous research (15–17), intraoperative blood loss > 500 ml was not significantly associated with postoperative organ/space SSI, which may be due to the prevalence of bloodless surgery. Other risk factors for SSI in previous studies, such as diabetes or perioperative hyperglycemia, obesity, low preoperative albumin and an immunosuppressive status (4, 6, 18–20), did not show any significant relationship with organ/space SSI in our results either. One possible reason is that diabetes and obesity may be more related to superficial SSI than to organ/space SSI. Moreover, according to the exclusion criteria, patients with a BMI ≥ 30 kg/m^2^ and those who were treated with glucocorticoids before surgery were not included, eliminating the potential influences of obesity and an immunosuppressive status. Additionally, because of the thorough consultation and treatment before elective surgery at PUMCH, the preoperative Glu and Alb levels for most patients in our study were within normal ranges.

The study also had several limitations. First, it was a secondary analysis based on two RCTs in a single center, which limited baseline variable selection and weakened their potential influences, so it cannot comprehensively reflect the real clinical situation. Second, the sample size was not large enough for subgroup analysis by different procedure types. Further study to investigate risk stratification in specific surgery type is necessary. Furthermore, our study was only a preliminary exploratory for risk stratification of organ/space SSI in advanced digestive system cancer and has not been validated in a wider population. A prospective validation cohort is needed to improve its generalization performance in the future.

## Conclusions

The risk stratification in this study provides a simple and convenient tool to identify the risk of postoperative organ/space SSI for patients with advanced digestive system cancer. Using this method, more targeted efforts can be implemented to detect or prevent organ/space SSI in a timely manner for those who have a greater risk. Further research is needed to investigate risk stratification in specific surgery type and validate the results in a wider population.

## Data Availability Statement

The datasets generated and analysed during the current study are not available because they were obtained from the hospital information system in our institution, which is not openly accessible to the public. However, the datasets could be available from the corresponding author on reasonable request.

## Ethics Statement

The studies involving human participants were reviewed and approved by the ethics committee of Peking Union Medical College Hospital. The patients/participants provided their written informed consent to participate in this study.

## Author Contributions

CS, LP, and YH contributed to the study design. CS, LP, and HG contributed to data acquisition. CS, LP, and YZ contributed to the data analysis. CS and LP wrote the report. All authors contributed to the article and approved the submitted version.

## Funding

This study was supported by the Chinese Academy of Medical Sciences Innovation Fund for Medical Sciences (2020-I2M-C&T-B-045), Peking Union Medical College Hospital Precipitation and Integration Foundation (ZC201906511) and Peking Union Medical College Hospital Science Foundation for Youths (pumch201912048). The funders had neither role in the design of the study, collection, analysis, interpretation of data, nor in writing the manuscript.

## Conflict of Interest

The authors declare that the research was conducted in the absence of any commercial or financial relationships that could be construed as a potential conflict of interest.

## Publisher’s Note

All claims expressed in this article are solely those of the authors and do not necessarily represent those of their affiliated organizations, or those of the publisher, the editors and the reviewers. Any product that may be evaluated in this article, or claim that may be made by its manufacturer, is not guaranteed or endorsed by the publisher.
